# SBRT treatment of abdominal and pelvic oligometastatic lymph nodes using ring‐mounted Halcyon Linac

**DOI:** 10.1002/acm2.13268

**Published:** 2021-05-25

**Authors:** Damodar Pokhrel, Aaron Webster, Joseph Stephen, William St Clair

**Affiliations:** ^1^ Medical Physics Graduate Program Department of Radiation Medicine University of Kentucky Lexington KY USA

**Keywords:** AcurosXB, Coplanar Geometry, FFF‐beam, Halcyon Linac, oligometastatic lymph nodes SBRT, single‐isocenter VMAT

## Abstract

**Purpose/objectives:**

This work seeks to evaluate the plan quality, treatment delivery efficiency, and accuracy of single‐isocenter volumetric modulated arc therapy (VMAT) of abdominal/pelvic oligometastatic lymph nodes (LNs) stereotactic body radiation therapy (SBRT) on Halcyon Linac.

**Materials and Methods:**

After completing the in‐house multitarget end‐to‐end phantom testing and independent dose verification using MD Anderson’s single‐isocenter/multi‐target (lung and spine target inserts) thorax phantom, eight patients with two to three abdominal/pelvic oligometastatic LNs underwent highly conformal single‐isocenter VMAT‐SBRT treatment using the Halcyon Linac 6MV flattening filter free (FFF) beam. Targets were identified using an Axumin PET/CT scan co‐registered with planning CT images and a single‐isocenter was placed between/among the targets. Doses between 25 and 36.25 Gy in 5 fractions were delivered. Patients were treated every other day. Plans were calculated in Eclipse with advanced AcurosXB algorithm for heterogeneity corrections. For comparison, Halcyon VMAT‐SBRT plans were retrospectively generated for SBRT‐dedicated TrueBeam with a 6MV‐FFF beam using identical planning geometry and objectives. Target coverage, conformity index (CI), dose to 2 cm away from each target (D2cm) and dose to adjacent organs‐at‐risk (OAR) were evaluated. Additionally, various treatment delivery parameters including beam‐on time were recorded.

**Results:**

Phantom measurements showed acceptable spatial accuracy of conebeam CT‐guided Halcyon SBRT treatments including compliance with MD Anderson’s single‐isocenter/multi‐targets phantom credentialing results. For patients, the mean isocenter to tumor center distance was 3.4 ± 1.2 cm (range, 1.5–4.8 cm). The mean combined PTV was 18.9 ± 10.9 cc (range, 5.6–39.5 cc). There was no clinically significant difference in dose to LNs, CI, D2cm and maximal doses to OAR between single‐isocenter Halcyon and Truebeam VMAT‐SBRT plans, although, Halcyon plans provided preferably lower maximal dose to adjacent OAR. Additionally, total monitor units, beam‐on time and overall treatment time was lower with Halcyon plans. Halcyon’s portal dosimetry demonstrated a high pass rate of 98.1 ± 1.6% for clinical gamma passing criteria of 2%/2 mm.

**Conclusion:**

SBRT treatment of abdominal/pelvic oligometastatic LNs with single‐isocenter VMAT on Halcyon was dosimetrically equivalent to TrueBeam. Faster treatment delivery to oligometastatic LNs via single‐isocenter Halcyon VMAT can improve clinic workflow and patient compliance, potentially reducing intrafraction motion errors for well‐suited patients. Clinical follow‐up of these patients is ongoing.

## 1 INTRODUCTION

Stereotactic body radiation therapy (SBRT) treatment of single or multiple abdominal and pelvic lymph nodes (LNs) is a fast, safe, and effective treatment option with one‐ and five‐year tumor local control rates of up to 100% and 70% and a low risk of treatment‐related toxicity.^1^, ^2^, ^3^, ^4^, ^5^, ^6^ In addition to providing higher therapeutic dose to multiple LNs, SBRT can reduce the number of patient hospital visits and will help to improve patient compliance and clinic workflow. Traditionally, LNs SBRT have been delivered using SBRT dedicated C‐arm Linac via conebeam CT image guidance or a robotic CyberKnife unit^3^, ^6^ and recently, utilizing volumetric modulated arc therapy (VMAT) for fast and effective treatment delivery.^7^, ^8^ Due to the advancement of MRI‐Linac technology, adaptive SBRT treatments to single or multiple LNs can be delivered in real time, significantly improving targeting accuracy.^9^, ^10^, ^11^, ^12^, ^13^, ^14^ However, MRI‐Linac treatments are very time consuming and not readily available to every patient’s cohort. Longer treatment times are very inconvenient for the patient (who is lying down in the treatment position), and can hinder clinic workflow.

For fast patient throughput, Varian recently introduced a fast‐rotating ring‐mounted Linac called the Halcyon V2.0 (Varian Medical Systems, Palo Alto, CA) platform for conventionally fractionated image‐guided radiation therapy (IGRT) treatments.^15^ This novel, but coplanar Linac was designed under tight performance specifications in order to improve patient safety and treatment delivery accuracy. In brief, the Halcyon V2.0 is equipped with a single‐energy 6MV‐FFF beam with a rapid gantry rotation speed of 4 revolutions per minute with a mean energy and nominal depth of maximal dose at 1.3 MeV and 1.3 cm respectively.^15^, ^16^, ^17^, ^18^, ^19^ In contrast with the SBRT‐dedicated C‐arm TrueBeam Linac, Halcyon Linac is equipped with newly designed double‐stacked and staggered 1 cm width MLC layers. The proximal and distal layers are offset by 5 mm allowing for a projected 5 mm effective MLC width at isocenter, like that of a standard SBRT‐dedicated TrueBeam Linac with Millennium 120 MLC. The jawless Halcyon Linac has a maximal field size of 28 × 28 cm^2^ with full MLC travel of 28 cm and is twice as fast as the standard Millennium 120 MLC. The stacked/staggered design allows for ultra‐low MLC leakage and transmission dose of <0.5%.^15^, ^16^, ^17^, ^18^, ^19^ Per machine specifications, Halcyon provides an improved penumbra with a smaller dosimetric leaf gap of approx. 0.1 mm. In addition to the MV‐conebeam CT imaging system, the Halcyon Linac is equipped with fast kilovoltage conebeam CT (kV‐CBCT) imaging that includes a high‐quality iterative CBCT reconstruction (iCBCT) algorithm for better online image quality.^20^, ^21^ This novel Linac is designed for a ‘one‐step patient set up and verification’ that automatically applies couch shifts for patient set‐up followed by the image‐guidance procedure for each treatment.^15^ Therefore, therapists do not need to manually apply the isocenter shifts inside the treatment room, significantly decreasing patient set‐up time and consequently reducing overall treatment time.

We have recently installed the Halcyon V2.0 in our center. Initial acceptance testing and commissioning results of the Halcyon Linac showed all acceptance and commissioning criteria met with the Varian’s specifications as described above.^15^, ^16^, ^17^, ^18^, ^19^, ^20^, ^21^ Due to the superior image quality, similar MLC width to standard Millennium MLC on TrueBeam linac, less leakage and transmission dose and the faster performance capabilities mentioned above, we have also commissioned Halcyon V2.0 for extracranial SBRT treatments following the SBRT protocol.^22^, ^23^ In addition to accounting for an SBRT board and Halcyon couch insert, we have commissioned the advanced AcurosXB algorithm in the Eclipse treatment planning system (TPS)^24^ (Varian Medical Systems, Palo Alto, CA) for more accurate accounting of heterogeneities corrections.

A few early Halcyon users reported fast and effective treatment delivery of conventionally fractionated breast, head and neck, and prostate treatments with similar plan quality when compared to C‐arm Linacs.^25^, ^26^, ^27^, ^28^ Additionally, Knutson et al. reported a retrospective dosimetric analysis of intracranial stereotactic radiation therapy (SRT) planning using the Halcyon Linac.^29^ In a study of 20 patients with a dose of 30 Gy in 5 fractions schemata, they showed that acceptable plan quality for brain SRT can be achieved using a coplanar Halcyon Linac. Another retrospective planning study by Li et al ^30^ recently demonstrated that the Halcyon V2.0 can generate plan quality similar to a stereotactic dedicated C‐arm linac for 6–10 intracranial lesions with diameter greater than 1.0 cm, using a single‐isocenter VMAT plan. While these retrospective planning studies demonstrate an acceptable plan quality, these plans were not used for the patient’s treatment on Halcyon Linac. In this report, in addition to phantom measurements and independent dose verification via MD Anderson’s multitarget thorax phantom credentialing, we evaluate eight consecutive patients’ plans with multiple LNs (2–3 nodes) who recently underwent SBRT treatments on our Halcyon Linac using a single‐isocenter VMAT plan. We evaluated for plan quality, treatment delivery efficiency and accuracy as part of commissioning and clinical implementation of our extracranial SBRT program on this novel ring‐mounted Halcyon Linac. Moreover, this report adds the benchmark study for other clinics to start extracranial SBRT on Halcyon including irradiating oligometastatic LNs.

## 2 MATERIALS AND METHODS

### 2.1 Phantom measurements and independent dose verification

For Halcyon Linac, to quantify the spatial positioning accuracy of a single‐isocenter/multitarget treatments as a function of tumor to isocenter distance, end‐to‐end phantom tests were performed. We have utilized 2 phantoms, provided with the Halcyon machine, that were designed to test the Halcyon linac performance. The first was the recently released QUART phantom (GmbH, Zorneding, Germany) with multiple high and low contrast inserts in it that were used to quantify the spatial resolution directly. A conebeam CT scan of the QUART phantom was obtained at the treatment position and the geometric accuracy was measured by 3D‐to‐3D matching with the planning CT images with respect to the multiple inserts at different locations. The second was the machine performance check (MPC) phantom on Halcyon that comes with 16 balls bearings (BBs) at the different locations up to 15 cm away from the center of the phantom. Similar to the QUART phantom, to further quantify the spatial localization accuracy as a function of distance to isocenter, a kV 3D/3D registration of the MPC phantom was performed by matching the BBs with the planning CT images. Moreover, the independent dose validation of the single‐isocenter/multitarget treatment on Halcyon was performed using the MD Anderson’s SBRT credentialing thorax phantom with two targets (spine and lung tumors with dosimetry system). This phantom was imaged, planned using a single‐isocenter VMAT, and irradiated with a SBRT prescription dose of 6.0 Gy in 1 fraction to both targets simultaneously for credentialing the NRG‐BR001 protocol.^23^ The tumor distance between the spine and lung targets was about 9 cm. Advanced Acuros‐based dose calculation was used. The phantom irradiation results were in compliance with the MD Anderson’s standards. Thus, after completing all the phantom validation testing, we are currently treating both conventionally fractionated and selected hypofractionated SBRT patients including abdominal/pelvic oligometastic LNs on our Halcyon Linac.

### 2.2 Patient cohort

After obtaining Institutional Review Board approval for our institute, eight consecutive oligometastatic abdominal and pelvic LNs patients who underwent SBRT treatments on ring‐mounted Halcyon Linac were selected for this study. These patients received 25–36.25 Gy in 5 fractions. All patients involved with this study had been diagnosed with nonmetastatic prostate cancer. In all patients, the primary prostate cancer was treated with local therapy including prostatectomy, radiation therapy and one patient treated with cryotherapy followed by salvage radiation therapy. Pathology of the original tumors ranged from Gleason score 3 + 4 (grade group 2) to Gleason score 4 + 5 (grade group 5). Follow‐up PSA evaluations revealed a rising trend. Patients were evaluated by Axumin PET/CT scans^31^, ^32^, ^33^ and were found to have two or more PET positive LNs. For all LNs patients, the evidence of two or more enlarged PET positive LNs metastases were found on Axumin PET/CT scans with no other sites of diseases.

### 2.3 CT simulation and target delineation

**Fig. 1:**
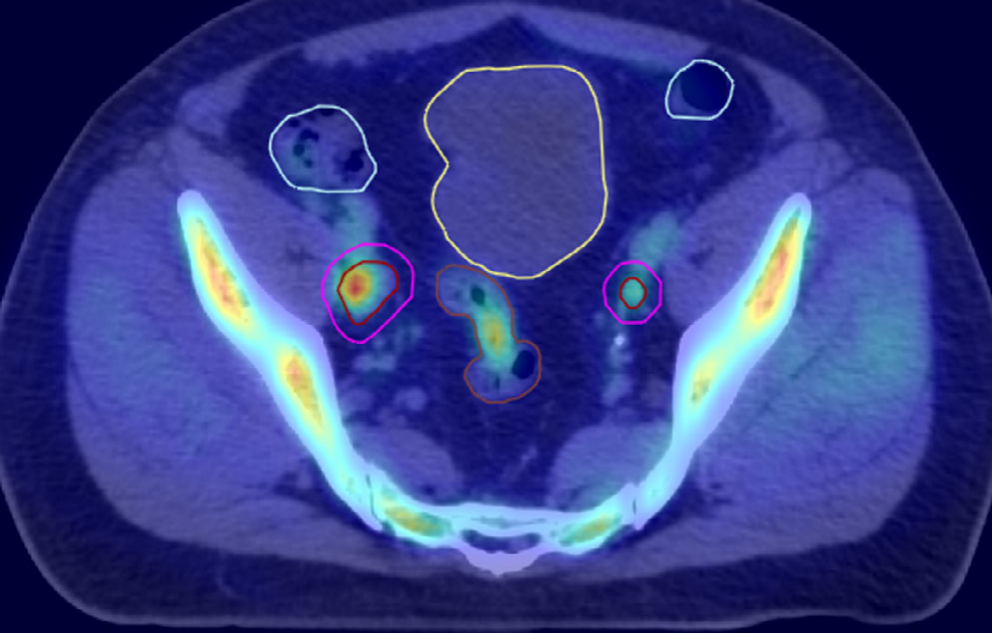
Co‐registered Axumin PET/CT scan with the planning CT images for locating the enlarged pelvic lymph nodes for example patient #6. Two‐bilateral iliac chain LNs were highlighted on Axumin scan.

All patients were immobilized using the Body Pro‐Lok^TM^ device (CIVCO system, Orange City, IA) in the supine position with their arms above the head. A knee cushion was used to immobilize the knees and legs. Patients were instructed to present for CT simulation and treatment with a comfortably full bladder and empty rectum. Patients were instructed not to urinate for one and a half to two hours before simulation and each fraction of SBRT treatment to help with daily patient set up reproducibility. To ensure a relatively empty rectum, patients were instructed to use miralax beginning 3 days prior to simulation and daily throughout the course of SBRT treatment. A free‐breathing 3D planning CT was acquired on a GE Lightspeed 16 slice CT scanner (General Electric Medical Systems, Waukesha, WI) with 512 × 512 pixels at 1.25 mm slice thickness in the axial helical mode. The 3D planning CT images were then imported into Eclipse TPS (Version 15.6) and co‐registered with the previously acquired Axumin PET/CT images (Fig. 1) for delineating each oligometastatic enlarged LN and designated these as the gross target volume (GTV). The planning target volume (PTV) was then generated by adding a uniform 5 mm margin around each GTV. Tumor characteristics for this patient cohort are summarized in Table 1. Each patient had 2–3 enlarged LNs on abdominal and pelvic sites. The combined PTV ranged from 5.57 to 39.5 cc (Table 1). Bladder and small bowel positioning are most important when targeting LNs in the pelvis. The OAR contours included duodenum, rectum, bladder, and small bowel for dose reporting.

**Table 1:** Main lymph nodes (LNs) characteristics of the patients included in this study.

Patient no.	No. of LNs involved	GTV of each LN (cc)	PTV of each LN (cc)	Combined PTV (cc)	Treatment site/LNs
I	2	1.46, 4.88	8.0, 16.71	24.71	Abdominal LNs
II	2	3.01, 1.67	14.02, 7.59	21.61	Periaotic LN
III	2	0.71, 0.25	4.85, 2.11	6.96	Abdominal LNs
IV	2	2.96, 2.13	23.31, 16.19	39.50	Iliac chain LNs
V	2	0.13, 0.41	1.68, 3.89	5.57	Para aortic LNs
VI	2	3.28, 1.60	13.47, 7.53	21.00	Iliac chain LNs
VII	2	3.07, 0.48	15.23, 3.09,	18.32	Para aortic LNs
VIII	3	0.82, 0.23, 0.38	7.03, 2.64, 3.38	13.05	Para aortic + Lt common iliac LNs

### 2.4 Clinical Halcyon VMAT plans

For all oligometastatic LNs patients, single‐isocenter VMAT‐SBRT plans were generated in the Eclipse TPS using 2 full arcs on a Halcyon Linac using a 6MV‐FFF (800 MU/min) beam. A single isocenter was placed between/among the targets and arcs had collimator angles automatically chosen for reducing the leakage dose to normal tissue between the targets. Prescription doses were 25 Gy to 36.25 Gy in 5 fractions to each target following the NRG‐BR001 protocol.^23^ All PTVs were planned with dose prescribed to the 90% isodose line and optimized such that 95% of each PTV received 100% of the prescription dose. The maximum dose to the PTV fell within each GTV. All clinical single‐isocenter VMAT plans were optimized with the Photon Optimizer (PO) MLC algorithm and the final dose calculation was performed with an advanced AcurosXB (Varian Eclipse TPS, Version 15.6) dose calculation algorithm^24^, ^34^, ^35^ on the 3D planning CT images with a 1.25 mm calculation grid size (CGS). The Halcyon couch was included in the final dose calculation. Dose to medium reporting mode and planning objectives were used for highly conformal target coverage, target dose homogeneity, reducing low‐ and intermediate dose spillage and minimizing dose to adjacent limiting OAR. These LN SBRT patients were treated every other day using Halcyon kV‐iCBCT image‐guidance protocol.

### 2.5 TrueBeam VMAT plans

For comparison, all clinical Halcyon LN SBRT patients’ plans were retrospectively reoptimized in the Eclipse TPS using the same number of full arcs, same collimator rotations and identical arc geometry including same isocenter location on our SBRT‐dedicated C‐arm TrueBeam Linac (Varian Medical Systems, Palo Alto, CA) equipped with a standard millennium 120 MLC and avoid the collision issue. For comparison, a similar 6MV‐FFF beam was used but with a maximal available dose rate setting of 1400 MU/min on TrueBeam. Furthermore, the Halcyon couch was removed and the TrueBeam couch was inserted into the plan. Optimization objectives, dose calculation algorithm, CGS, dose reporting, convergence mode, and PO MLC optimizer used for the SBRT‐dedicated TrueBeam SBRT plans were identical to the Halcyon plans. However, TrueBeam VMAT plans utilized the jaw‐tracking option during plan optimization to further minimize the out‐of‐field dose that was not available on jawless Halcyon Linac. All TrueBeam VMAT‐SBRT plans’ PTV coverage was normalized identically to clinical Halcyon VMAT plans providing similar hotspots in the targets.

### 2.6 Plan comparison

The dose‐volume histogram (DVHs) and isodose curves of Halcyon and TrueBeam VMAT plans were compared. The original clinical single‐isocenter Halcyon VMAT and reoptimized TrueBeam VMAT plans were compared for target conformity, dose to GTV nodes and maximal dose 2 cm away from each target (D2cm). The maximal dose to immediately adjacent OAR were evaluated for duodenum, small bowel, rectum, and bladder. Distance to isocenter was determined by finding the coordinates of each PTV geometric center and calculating Euclidian distance in 3D geometry with the isocenter coordinates. Moreover, treatment delivery efficiency and accuracy was documented by recording the total number of monitor units (MU) per fraction and the ratio of total number of MU per fraction to the prescription dose in cGy is defined as the modulation factor (MF). The beam‐on time (BOT) was recorded during the delivery of the quality assurance (QA) plan at both machines. For each patient, BOT was added to patient set up and verification time, CBCT scanning and image matching time for each machine, and estimated overall treatment time. Dosimetric verification of these plans was performed using the portal dosimetry (PD) measurement QA procedure following the previously established guidelines.^36^, ^37^, ^38^ A gamma evaluation criteria of 2%/2mm with a low dose threshold set to 5% was used. The electronic portal‐imaging device (EPID, aS1200 flat panel detector, Varian Medical Systems, Palo Alto, CA) mounted on the Truebeam and Halcyon Linacs were used for measurement. This detector has an active area of 400 mm × 400 mm with a high‐resolution pixel size of 0.34 mm. The mean and standard deviation (range) for each dose metric was compared for all dosimetric parameters, target coverage, OAR doses, and treatment delivery parameters. Statistical analysis was performed using the Microsoft Excel (Microsoft Corp, Redmond WA) program. Mean, standard deviation (STD), and range values for each of the dosimetric parameters were compared for both plans.

## 3 RESULTS

### 3.1 Phantom measurements

On these phantom measurements, it has been observed that CBCT‐guided geometric accuracy on our Halcyon linac was within 1 mm (average, 0.76 mm) at 5 cm and less than 1.3 mm (average, 0.86 mm) at 10 cm distance from the isocenter, respectively. This suggests that while using kV 3D/3D matching on Halcyon, the patient set up accuracy of about ±1 mm can be achieved at 10 cm away from the isocenter. Additionally, credentialing results of MD Anderson’s anthropomorphic thorax phantom incorporating dosimetry inserts in the lung‐equivalent and spine tumors (with TLD and films located in the targets and OAR) satisfied both the TLD and film dosimetric requirements established by the IROC for SBRT treatments of single‐isocenter/multitargets setting on Halcyon. In this measurement, the average TLD and film measurement results were within ±2.0% and 96% gamma index over all three planes respectively.

### 3.2 Patient’s plans

#### 3.2.1 GTV nodes and PTV coverage

Table 2 summarizes the target coverage, conformity index and intermediate dose‐spillage for both plans. For the identical target coverage, Halcyon VMAT plans were more conformal and provided slightly less intermediate dose spill shown by lower values of D2cm, compared to SBRT‐dedicated TrueBeam VMAT plans (see Table 2). Similar relative dose to the GTVs was obtained for both plans.

**Table 2:** Evaluation of target coverage and conformity, intermediate dose‐spillage and GTV nodal doses for all 8‐LNs SBRT patients (17 nodes) for both plans.

	Parameters	Halcyon VMAT	TrueBeam VMAT
Combined PTV	Volume covered by Rx dose (%)	95.0	95.0
	CI	1.05 ± 0.1 (0.99–1.15)	1.07 ± 0.1 (0.98–1.22)
Intermediate dose‐spillage	D2cm	50.5 ± 6.8 (39.5–58.7)	51.0 ± 6.9 (42.0–60.2)
GTV coverage (n = 17)	Minimum dose (%)	101.1 ± 1.4 (99.7–104.0)	100.6 ± 2.2 (97.8–104.9)
	Maximum dose (%)	108.3 ± 1.4 (106.0–110.5)	108.2 ± 2.5 (105.5–112.5)
	Mean dose (%)	104.3 ± 1.1 (103.2–105.8)	104.0 ± 1.8 (102.0–107.5)

Dose was 25 Gy to 36.25 Gy in 5 fractions to each abdominal/pelvic LN. Mean ± SD (range) was reported.

SD, standard deviation; n, no. of GTV.

#### 3.2.2 Dose to adjacent OAR

Table 3 shows the number of LNs per patient basis, their prescription dose and the average 3D Euclidian distance between the targets. The isocenter to tumor center distance for each patient ranged from 1.5 cm to 4.8 cm, all less than 5 cm. Table 3 also shows immediately adjacent OAR and the maximal dose to the OAR achieved for the both plans. In this cohort, for each patient, the Halcyon VMAT provided similar but relatively lower maximal dose to OAR compared to TrueBeam VMAT plans (Table 3), systematically suggesting better plan quality.

**Table 3:** Analysis of the maximal dose to immediately adjacent critical structures including the average distance between target center and isocenter on a per‐patient basis.

Patient no.	No. of LNs involved	Prescribed dose to each PTV	Avg. distance to isocenter (cm)	Dose limiting OAR	Maximal dose to adjacent OAR	
					Halcyon VMAT (Gy)	TrueBeam VMAT (Gy)
I	2	7.25 Gy × 5	4.8	Small bowel	23.11	23.35
				Bladder	33.45	34.14
II	2	6.0 Gy × 5	3.7	Small bowel	31.43	32.14
III	2	7.25 Gy × 5	1.5	Small bowel	23.11	23.70
IV	2	7.0 Gy × 5	4.1	Rectum	12.37	12.30
				Small bowel	15.18	16.01
V	2	5.0 Gy × 5	1.8	Small bowel	25.23	25.14
VI	2	6.0 Gy × 5	3.5	Small bowel	21.72	22.13
				Bladder	17.33	16.47
				Rectum	18.21	18.58
VII	2	7.25 Gy × 5	2.9	Small bowel	19.45	19.65
				Duodenum	22.18	22.05
				Spinal cord	9.49	10.12
VIII	3	7.25 Gy × 5	4.5	Small bowel	32.81	32.25

#### 3.2.3 Example case

**Fig. 2:**
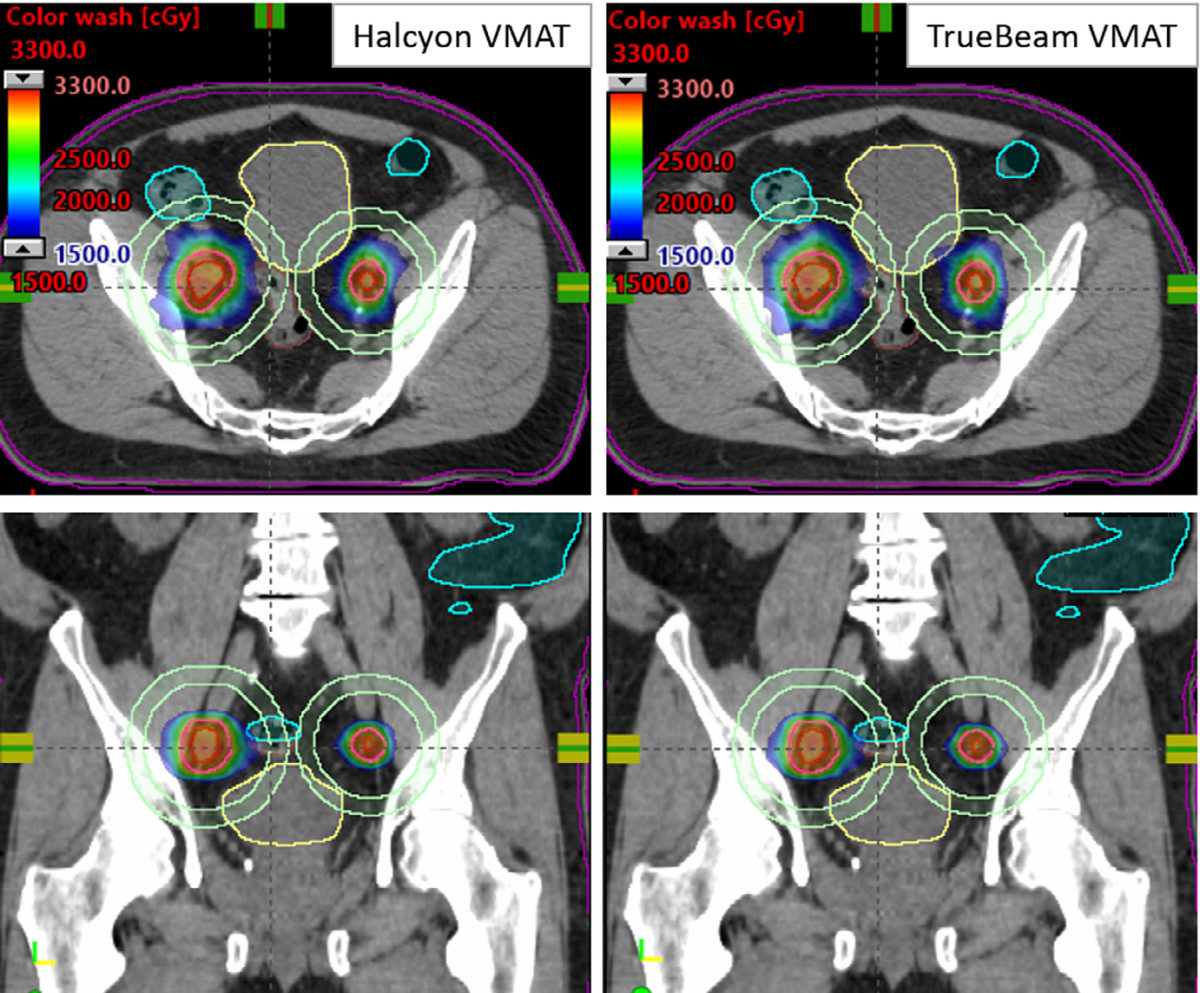
SBRT isodose colorwash through the isocenter location (cross‐hair) for the clinical Halcyon VMAT (left panel) vs TrueBeam VMAT plan (right panel) for an example patient #6 with bilateral iliac LNs. Dose was 30 Gy in 5 fractions to each lesion. A few critical structures shown are rectum (brown), bladder (yellow), and sigmoid (light blue). The D2cm (light green) rings are shown for each LN.

Figure 2 shows an example (case #6) of the original clinical single‐isocenter Halcyon vs TrueBeam VMAT plan of a patient who presented with 2 bilateral iliac chain LNs. Dose distributions are displayed in axial and coronal views (50%–110%, dose colorwash of 30 Gy in 5 fractions). Highly conformal and similar isodose distribution was obtained for both plans.

**Fig. 3:**
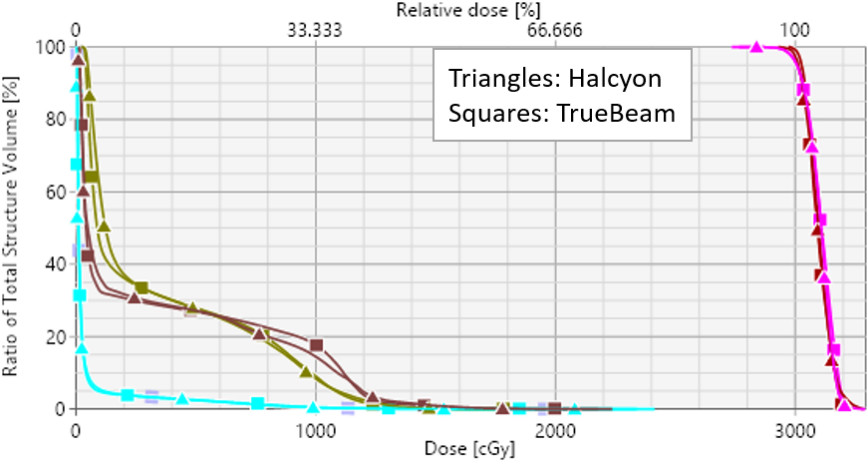
The DVH parameters for both plans shown in Fig. 2. Identical target coverage to the combined PTV (pink), and combined GTV nodes (dark brown) and similar OAR sparing were achieved on both plans.

Figure 3 can be analyzed by their respective DVH for target coverage, GTV dose and dose to OAR. For identical combined PTV coverage, the clinical Halcyon VMAT (triangles) and the corresponding TrueBeam VMAT (squares) plans provided similar GTV doses and dose to the immediately adjacent OAR (brown, rectum; yellow bladder and light blue, small bowel). Similar dosimetric indices were achieved for both plans.

#### 3.2.4 Treatment delivery efficiency and accuracy

Treatment delivery efficiency was evaluated by comparing the total number of MU and BOT was estimated by recording portal dosimetry quality assurance (QA) delivery time on both machines. Compared to clinical Halcyon VMAT, TrueBeam VMAT plans delivered slightly higher total MU, 124 on average, corresponding to a relatively higher beam MF. Mean values of total MU and MF were 2369 and 3.55 for clinical single‐isocenter Halcyon VMAT plans vs 2490 and 3.74 for the corresponding TrueBeam VMAT plans (Table 4). For the single‐isocenter VMAT plans, a small value of MLC modulation with Halcyon Linac may be desirable as it may further reduce MLC leakage dose. This may add to the benefit of treating oligometastatic LNs SBRT patients on Halcyon instead of a traditional C‐arm linac. BOT and overall treatment time for the clinical Halcyon VMAT vs TrueBeam VMAT plans are shown in Table 4 as well. For the maximum dose rate settings of 800 MU/min (on Halcyon) and 1400 MU/min (on TrueBeam) on both machines, the mean overall treatment time on Halcyon was still 2.12 min (range, 1.4–2.5 min) faster than TrueBeam VMAT plans. This is due to multiple reasons. One is due to the faster patient set up and verification time on Halcyon’s built in “one‐step patient set up” process and faster kV‐iCBCT scanning and auto images matching procedure; compared to slow CBCT scanning (1 min) and manual image‐matching on TrueBeam Linac. The other reason is the four‐times faster gantry rotation speed of Halcyon compared to the Truebeam Linac. Additionally, for these prescription dose ranges, Truebeam exhibited longer treatment times due to its inability to achieve its maximum dose rate of 1400 MU/min for all control points resulting from relatively higher beam modulation and gantry rotation speed limitations. This suggests a shorter overall treatment time that a patient is on the Halcyon couch for SBRT treatment of multiple LNs, potentially minimizing intrafraction motion errors.

**Table 4:** Comparison of average values of treatment delivery parameters (SD and range) between the clinical single‐isocenter Halcyon VMAT and corresponding re‐planned TrueBeam VMAT plans for all 8‐LNs SBRT patients treated on Halcyon Linac.

Beam delivery parameters	Halcyon VMAT	TrueBeam VMAT
Total monitor units (MU)	2369 ± 772 (1476–3860)	2490 ± 741 (1576–3910)
Modulation factor (MF)	3.55 ± 0.93 (2.5–5.5)	3.74 ± 0.89 (2.7–5.6)
BOT (min)	2.96 ± 0.97 (1.8–4.8)	2.41 ± 0.62 (2.1–3.39)
Treatment time (min)	7.96 ± 0.97 (6.8–9.8)	10.08 ± 0.62 (9.3–11.3)
Pretreatment PD QA pass rates, [2%/2mm] (%)	98.1 ± 1.6 (96.9–100.0)	97.7 ± 1.8 (96.6–100.0)

SD, standard deviation.

As described above, treatment delivery accuracy of the single‐isocenter VMAT‐SBRT plans for multiple LNs was evaluated by delivering each plan in QA measurement mode to both Linacs using an on‐board EPID imager and analyzing the gamma pass rates via the PD QA procedure.^36^, ^37^, ^38^ We have compiled SBRT QA pass rates following the TG‐218 criteria recommended on pre‐treatment QA tolerance level (overall pass rate ≥95% at a γ– criteria of 2%/2mm with low threshold of 5%).^38^ With PD QA, all plans satisfied this criterion and were similar on both machines. The dose delivery accuracy of the single‐isocenter Halcyon VMAT and the corresponding TrueBeam VMAT plans were 98.1 ± 1.6% (range 96.9–100.0%) and 97.8 ± 1.8% (range 96.6–100%) respectively.

**Fig. 4:**
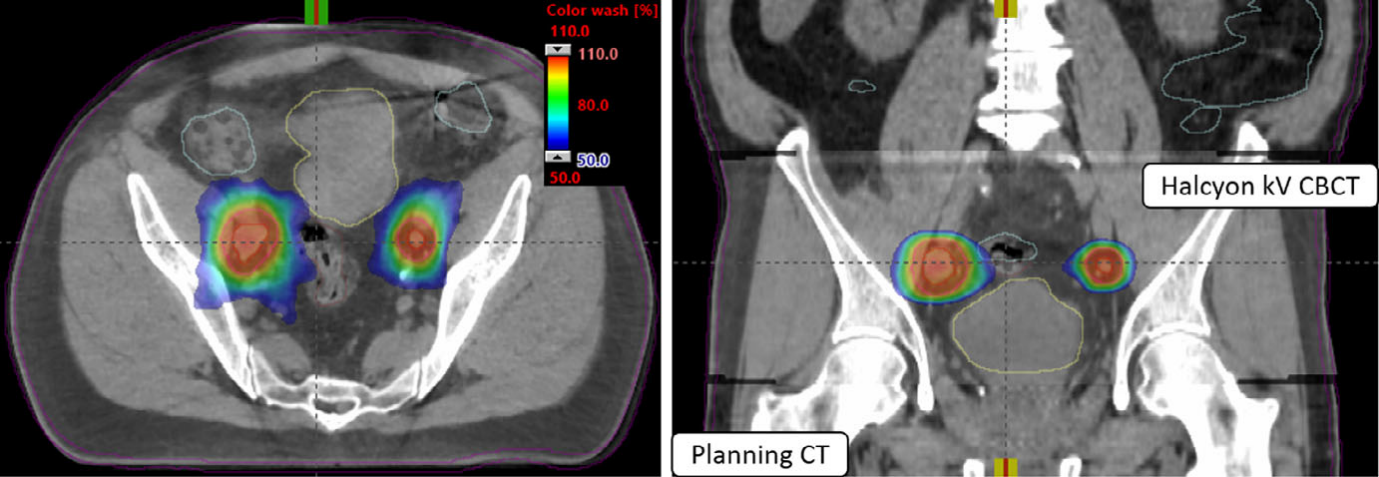
Axial and coronal views of Halcyon kV‐CBCT images (see inset in the coronal view) co‐registered with planning CT images used for image‐guided single‐isocenter (see, cross‐hair) VMAT‐SBRT treatment to both lesions on Halcyon is shown. In addition to anatomical landmarks, the planned dose colorwash (50%–110% isodose cloud) is overlaid for this treatment to demonstrate the delivery accuracy. Halcyon kV‐CBCT images were acquired in the treatment position in free breathing and rigid‐registration was performed via automatic image‐registration mode on Halcyon followed by manually fine‐tuning the registration for better alignment of both nodes via soft‐tissue alignment.

Figure 4 shows the patient set up and verification on Halcyon kV‐CBCT images of the same example patient #6. The planned isodose color wash is superimposed with the daily Halcyon kV‐CBCT images after the three degrees‐of‐freedom (3DoF) translational couch corrections were applied. This patient was initially positioned using external marks and in‐room blue lasers, followed by the “one‐step patient set up” and a 15‐s pretreatment iterative kV‐CBCT scan being obtained. Halcyon kV‐CBCT pelvis imaging protocol parameters (125 kV, 1080 mAs) were used with 512 × 512 pixels and 2.0 mm slice thickness for iCBCT reconstruction. An in‐house SBRT/IGRT protocol was applied registering the fast pretreatment kV‐iCBCT with the planning CT scans (see Fig. 4). For each treatment, rigid‐registration was performed automatically based on region of interest and bony landmarks followed by a manual refinement of the soft‐tissues matching and confirmed by the treating physician and physicist for the alignment of both LNs. The patient was then repositioned by applying the 3DoF couch corrections from the original isocenter and the treatment was delivered. Our image‐guided SBRT protocol limits translational 3DoF couch corrections to less than ±3.0 mm, on average in each direction for all LNs SBRT treatments.

## 4 DISCUSSION

After completing the in‐house end‐to‐end tests with phantoms measurements and independent dose verification via credentialing the MD Anderson’s single‐isocenter/multi‐targets thorax phantom irradiation, in this study we have presented our initial clinical experiences using Halcyon Linac for fast, effective and accurate planning and delivery of single‐isocenter VMAT plans for SBRT treatment of multiple oligometastic abdominal/pelvic LNs. Our single‐isocenter VMAT plans on Halcyon for SBRT of multiple LNs uses two full coplanar arcs with stacked/staggered MLC and automatically selected patient specific collimator angles to minimize leakage dose from MLC travelling in between the targets. Single‐isocenter VMAT‐SBRT plans were highly conformal and achieved adequate target coverage (see Table 2 for dose to GTV nodes, CI and D2cm) compared to SBRT‐dedicated TrueBeam plans. For all patients, the single‐isocenter Halcyon VMAT plans provided similar or better plan quality with slightly lower maximal dose to the adjacent OAR (Table 3). Due to the relatively faster patient set up, verification and treatment delivery workflow, the single‐isocenter Halcyon treatment was well‐tolerated by all patients. The average overall treatment time (door‐to‐door) was less than 10 min per fraction. The average VMAT‐SBRT QA gamma passing rates of 98.1% (2%/2mm clinical gamma passing criteria) demonstrates an excellent potential for fast, reliable, and accurate delivery of a single‐isocenter VMAT‐SBRT treatment on Halcyon Linac for multiple oligometastatic LNs synchronously.

Recently, to improve patient compliance and treatment delivery efficiency, SBRT treatment of multiple synchronous extracranial lesions using a single‐isocenter plan has been an active area of research.^23^, ^39^, ^40^, ^41^, ^42^, ^43^, ^44^, ^45^ For instance, Pokhrel et al. treated 14 synchronous lung SBRT patients with 2–5 lesions using single‐isocenter VMAT plans on TrueBeam Linac with 6MV‐FFF (1400 MU/min) beam.^45^ The patients’ prescription dose was 50 Gy and 54 Gy in 5 and 3 fractions to each lesion with an average beam on time of 3.5 min/fraction. Average isocenter to tumor distance was 5.6 cm. In this cohort, early clinical follow‐up results (median, 9 months) indicate that tumor local‐control rate was 100% with no reported treatment‐related lung or ribs toxicity. Similar to this cohort report, utilizing a 6MV‐FFF beam on Halcyon Linac our single‐isocenter VMAT plans delivered fast (average, 2.96 min BOT), accurate and effective treatment to multiple oligometastatic abdominal/pelvic LNs SBRT.

Although we have demonstrated a clinically useful and patient friendly single‐isocenter VMAT‐SBRT approach to multiple abdominal and pelvic LNs on Halcyon Linac, there are some limitations of this study. First, for multiple LNs SBRT patients, there is currently no way to apply rotational couch corrections on Halcyon Linac. For patient set up verification, there are presently only three translational corrections that can be applied. On the other hand, our TrueBeam Linac is capable of applying both rotational and translational couch corrections and may better align multiple lesions on a single daily CBCT scan and minimize the patient set up errors in the treatment of LNs SBRT. A few recent studies have shown loss of target coverage due to rotational set up errors in a single‐isocenter/multilesions setting.^46^, ^47^, ^48^ However, the exact magnitude of dosimetric discrepancy in using six‐degrees‐of‐freedom (6DOF) couch corrections for treating multiple LNs SBRT treatment on Halcyon is not yet known. In the future, we plan to quantify the dosimetric impacts of rotational patient set up corrections on Halcyon Linac for multilesion SBRT treatments including single‐isocenter multiple VMAT LNs SBRT. The second issue with the current Halcyon V2.0 is that Halcyon’s highest achievable maximal dose‐rate of 800 MU/min that was achieved for all control points. Although, Halcyon V2.0 presents a similar FFF beam profile to the TrueBeam Linac’s 6MV‐FFF beam; however, Halcyon’s highest dose‐rate of 800 MU/min (that was achieved for all control points in these clinical VMAT plans) could significantly affect the beam‐on time compared to a maximal achievable dose rate of 1400 MU/min on TrueBeam Linac. Although for these TrueBeam VMAT plans (for these prescription doses), the maximal dose rate of 1400 MU/min did not achieve for all control points. In addition, the overall treatment time was limited by the gantry rotation speed. We estimate that increasing maximal dose rate to 1000 MU/min on Halcyon Linac could potentially further improve the treatment delivery efficiency of multilesions VMAT‐SBRT treatments.

Briefly, including phantoms measurements and independent dose validation, all Halcyon single‐isocenter VMAT‐SBRT plans to multiple synchronous LNs treatment were evaluated, thoroughly comparing the corresponding SBRT‐dedicated TrueBeam plans as listed in Tables 2, 3, 4. All treatment delivery parameters were acceptable and deemed high quality plans for treating multiple LNs SBRT patients on Halcyon with an overall faster treatment time of less than 10 min, potentially benefiting the patients who cannot lie down flat in the treatment position for longer treatment time and potentially minimizing inter‐fraction/intrafraction motion errors.^49^ However, clinical follow‐up results of tumor local‐control and treatment related toxicities are necessary to confirm patient outcomes and are ongoing in our center. Many researchers are currently investigating adaptive treatments to single‐ and multiple LNs SBRT using MRI‐linac.^9^, ^10^, ^11^, ^12^, ^13^, ^14^ First of all, MRI‐Linac systems are costly and are not readily available for every patient cohort. Second, even for a fully dedicated SBRT team, MRI‐Linac treatments are very complex and require much longer treatment times (in hours). Patients may not be able to tolerate those treatments due to back pain and nervousness, possibly resulting in incomplete SBRT treatments. Moreover, a systematic study by Winkel et al.^11^ demonstrated that there was no significant difference between the GTV coverage and mean GTV dose between the MRI‐Linac vs conventional fast co‐planner VMAT plans on CBCT‐Linac treatment. They argue that the justification of online MRI‐guided radiotherapy for these patients with five fractions schemata was questionable on the present MRI‐Linac. In addition to reducing intrafraction motion errors, shortening overall treatment time using co‐planner VMAT plans can improve patient comfort and compliance and improve safety and clinic throughput. Based on this research and other previous studies,^29^, ^30^ we plan to expand our Halcyon Linac service to treat other extracranial disease sites to selected patients with two vertebral body lesions, or multilesion liver and lung SBRT patients. Because of the lower total MU per fraction (relatively shorter BOT) and a fast 15‐s kV‐iCBCT scanning time (equivalent to 1 breath‐hold), deep inspiration breath‐hold SBRT treatments to extracranial thoracic or abdominal/pelvic lesions on Halcyon Linac merit future investigation. In addition to Axumin PET/CT registration, going forward we plan to use 4D‐CT imaging for further confirming the enlarged LNs for multitarget delineation.

## 5 CONCLUSION

In this report, we have demonstrated the treatment planning feasibility, delivery efficiency and accuracy, and clinical implementation of single‐isocenter VMAT‐SBRT treatment to the abdominal/pelvic oligometastastic LNs on ring‐mounted Halcyon Linac. The results of this study demonstrate that treatment of multiple LNs SBRT patients on the fast‐rotating Halcyon Linac is safe, effective, accurate, and clinically comparable to the SBRT‐dedicated TrueBeam Linac. For those clinics equipped with a Halcyon Linac only or the centers with high volume SBRT patients, commissioning and SBRT treatment of multiple LNs is suggested, therefore this fast‐rotating Halcyon Linac can provide access to standardized, fast and curative SBRT treatment for the large and underserved cohort of multiple extracranial lesions cancer patients and improve the clinic workflow. Clinical outcomes of tumor local‐control rates and treatment related toxicity of the patients who underwent single‐isocenter/multiple LNs SBRT treatments on Halcyon Linac is underway. Further investigation of dosimetric impacts of rotational corrections on a single‐isocenter/multilesions VMAT‐SBRT setting on Halcyon Linac is warranted.

## CONFLICT OF INTEREST

The authors have no relevant conflict of interest to disclose.

## Author contributions

DP conceived the project. DP, AW, and JS collected and analyzed the data. WSC provided clinical expertise and definitive supervision of the paper. DP drafted the preliminary manuscript and all co‐authors revised and approved the final manuscript for submission.

## Data Availability

Research data are not shared.
